# *iMeta* Conference 2025: Creating high‐impact international journals

**DOI:** 10.1002/imt2.70086

**Published:** 2025-10-15

**Authors:** Zhihao Zhu, Lin Zhang, Xiaofang Yao, Meiyin Zeng, Yao Wang, Hao Luo, Yuanping Zhou, Tianyuan Zhang, Jiani Xun, Defeng Bai, Haifei Yang, Shanshan Xu, Yang Zhou, Yunyun Gao, Jinbo Xu, Wei Han, ZiAng Shen, Bangzhou Zhang, Tengfei Ma, Xiu‐Lin Wan, Chuang Ma, Fengjiao Hui, Hao Bai, Lijing Bai, Qing Bai, Qiangguo Bao, Guodong Cao, Peng Cao, Qiqi Cao, Hu Chen, Jiawen Chen, Jiaxu Chen, Lihua Chen, Tingting Chen, Yi Chen, Haipeng Cui, Shaoxing Dai, Xi‐Jian Dai, Xiaofeng Dai, Yanqi Dang, Lei Deng, Yun Deng, Xia Ding, Binhua Dong, Ling Dong, Shujie Dou, Hongzhi Du, Zhencheng Fang, Xiaoxiao Feng, Min Fu, Yuan Gao, Wenping Gong, Xiang Guo, Wenjie Han, Zikai Hao, Zheng‐Guo He, Haibo Hu, Haiming Hu, Xuefei Hu, Liang Huang, Xianya Huang, Xueting Huang, Haochen Hui, Dingjiacheng Jia, Aimin Jiang, Di Jiang, Kun Jiang, Dewei Jiang, Ying Jin, Kunyang Lai, Chun Li, Feng Li, Fuyong Li, Jing Li, Juan Li, Junling Li, Kui Li, Ling Li, Moli Li, Peiwu Li, Peng Li, Runze Li, Shengnan Li, Shujin Li, Wanting Li, Wenting Li, Xiaojing Li, Xinrui Li, Xuemeng Li, Qiqi Liang, Xiaoping Liao, Boyang Liu, Canzhao Liu, Chang Liu, Duanrui Liu, Furong Liu, Jianjun Liu, Jinyao Liu, Siqi Liu, Tianyang Liu, Wenjuan Liu, Yan Liu, Yang Liu, Yi Liu, Yuan Liu, Yunhuan Liu, Zhipeng Liu, Zhiyong Liu, Xin Lu, Xiao Luo, Guanju Ma, Jialin Meng, Yuanfa Meng, Runyu Miao, Linxuan Miao, Yawen Ni, Dongze Niu, Tingting Niu, Hongzhao Pan, Guoqiang Qin, Tiantian Qiu, Yueping Qiu, Hui Qu, Linghang Qu, Na Ren, Qiang Sun, Run Shang, Peize She, Xihui Shen, Bohan Shi, You Shu, Jiawei Song, Weibin Song, Qi Su, Qingzhu Sun, YuPing Sun, Zijin Sun, Bufu Tang, Deqin Tang, Hua Tang, Yongfu Tao, Teng Teng, Yanye Tu, Cheng Wang, Hui Wang, Yunhao Wang, Chunli Wang, Dingjie Wang, Gang Wang, Jin Wang, Kaiyi Wang, Mingbang Wang, Shan Wang, Shixiang Wang, Xiaojie Wang, Xing‐Chang Wang, Yunzhe Wang, Jiale Wang, Zheng Wang, Weijie Wang, Yongjun Wei, Wei Xu, Fan Wu, Junling Wu, Shijuan Wu, Jian Xiao, Weihua Xiao, Yang Xiao, Xi Xiong, Xue Xiong, Feng Xu, Junyu Xu, Wen Xu, Jun Xu, Yao Xu, Jun Yan, Lutian Yao, Jia Yang, Lulu Yang, Xingzhen Yang, Naiyi Yin, Hua You, Min You, Ting Yu, Yongyao Yu, Renqiang Yu, Shuofeng Yuan, Chaoxiong Yue, Xiaoya Zeng, Andong Zha, Leilei Zhai, Chi Zhang, Dong Zhang, Hengguo Zhang, Heng Zhang, Hongyu Zhang, Jiahao Zhang, Jinyang Zhang, Lishan Zhang, Qi Zhang, Xiang Zhang, Xiangyu Zhang, Xuelei Zhang, Yancong Zhang, Yuan Zhang, Zhenyu Zhang, Jiwei Zhao, Jingxuan Zhao, Kai Zhao, Mingjuan Zhao, Yi Zhao, Yunxiang Zhao, Jixin Zhong, Ling Zhong, Xiangjian Zhong, Dan Zhou, Wei Zhou, Wen Zhou, Yiqian Zhou, Zhemin Zhou, Shiquan Zhu, Shuang‐Jiang Liu, Suyin Feng, Shuangxia Jin, Chuanxing Xiao, Ziheng Wang, Peng Luo, Tong Chen, Gang Chen, Yong‐Xin Liu

**Affiliations:** ^1^ Genome Analysis Laboratory of the Ministry of Agriculture and Rural Affairs, Agricultural Genomics Institute at Shenzhen Chinese Academy of Agricultural Sciences Shenzhen China; ^2^ Zhanjiang Key Laboratory of Human Microecology and Clinical Translation Research, School of Basic Medical Sciences Guangdong Medical University Zhanjiang China; ^3^ Hubei Shizhen Laboratory, School of Basic Medical Sciences Hubei University of Chinese Medicine Wuhan China; ^4^ Zhanjiang Key Laboratory of Human Microecology and Clinical Translation Research, the Marine Biomedical Research Institute, College of Basic Medicine Guangdong Medical University Zhanjiang China; ^5^ College of Life Sciences Qingdao Agricultural University Qingdao China; ^6^ Anhui Academy of Agricultural Sciences Institute of Agro Products Processing Hefei China; ^7^ Oil Crops Research Institute of the Chinese Academy of Agricultural Sciences Wuhan China; ^8^ School of Ecology and Nature Conservation Beijing Forestry University Beijing China; ^9^ Inner Mongolia Key Laboratory of Dairy Biotechnology and Engineering Inner Mongolia Agricultural University Hohhot Inner Mongolia China; ^10^ College of Food Science and Engineering Central South University of Forestry and Technology Changsha China; ^11^ School of Biomedical Sciences and Engineering South China University of Technology Guangzhou China; ^12^ Xiamen Treatgut Biotechnology Co., Ltd Xiamen China; ^13^ State Key Laboratory of Herbage Improvement and Grassland Agro‐Ecosystems, Centre for Grassland Microbiome, College of Pastoral Agriculture Science and Technology Lanzhou University Lanzhou China; ^14^ College of Life Sciences Northwest A&F University Yangling China; ^15^ National Key laboratory of Crop Gentic Improement, Hubei Hongshan Laboratory, College of Plant Science and Technology Huazhong Agricultural University Wuhan China; ^16^ Laboratory of Radiation Medicine, West China School of Basic Medical Sciences & Forensic Medicine Sichuan University Chengdu China; ^17^ Shenzhen Branch, Guangdong Laboratory for Lingnan Modern Agriculture, Genomics Institute at Shenzhen Chinese Academy of Agricultural Sciences Shenzhen China; ^18^ Key Laboratory of Anhui Province for Emerging and Reemerging Infectious Diseases University of Science and Technology of China Hefei China; ^19^ State Key Laboratory for Quality Ensurance and Sustainable Use of Dao‐di Herbs, National Resource Center for Chinese Materia Medica China Academy of Chinese Medical Sciences Beijing China; ^20^ The First Affiliated Hospital Anhui Medical University Hefei China; ^21^ Nanjing University of Chinese Medicine Nanjing China; ^22^ National Key Laboratory of Veterinary Public Health and Safety, College of Veterinary Medicine China Agricultural University Beijing China; ^23^ Wuhan Benagen Technology Co., Ltd Wuhan China; ^24^ Guangzhou University of Chinese Medicine Guangzhou China; ^25^ School of Chinese Medicine Beijing University of Chinese Medicine Beijing China; ^26^ Institute of Chinese Materia Medica China Academy of Chinese Medical Sciences Beijing China; ^27^ First College for Clinical Medicine Nanjing University of Chinese Medicine Nanjing China; ^28^ Department of Gastroenterology and Hepatology The First Medical Center, Chinese PLA General Hospital Beijing China; ^29^ School of Basic Medical Sciences Southwest Medical University Luzhou China; ^30^ Institute of Primate Translational Medicine Kunming University of Science and Technology Kunming China; ^31^ Department of Radiology, The Second Affiliated Hospital, Jiangxi Medical College Nanchang University Nanchang China; ^32^ The First Affiliated Hospital of Xi'an Jiaotong University, Xi'an Jiaotong University Xi'an China; ^33^ Institute of Digestive Diseases, China‐Canada Center of Research for Digestive Diseases, Longhua Hospital Shanghai University of Traditional Chinese Medicine Shanghai China; ^34^ Shanghai Veterinary Research Institute, Chinese Academy of Agricultural Sciences Shanghai China; ^35^ Institute of Hydrobiology, Chinese Academy of Sciences Wuhan China; ^36^ School of Life Sciences Nanchang University Nanchang China; ^37^ Laboratory of Gynecologic Oncology, Fujian Maternity and Child Health Hospital, College of Clinical Medicine for Obstetrics & Gynecology and Pediatrics Fujian Medical University Fuzhou China; ^38^ M20 Genomics Hangzhou China; ^39^ Hebei Key Laboratory of Forensic Medicine, Hebei Collaborative Innovation Center of Forensic Medical Molecular Identification, College of Forensic Medicine Hebei Medical University Shijiazhuang China; ^40^ School of Pharmacy Hubei University of Chinese Medicine Wuhan China; ^41^ Microbiome Medicine Center, Department of Laboratory Medicine, Zhujiang Hospital Southern Medical University Guangzhou China; ^42^ The Rural Development Academy, Zhejiang University Hangzhou China; ^43^ Anhui Province Key Laboratory of Integrated Pest Management on Crops, College of Plant Protection Anhui Agricultural University Hefei China; ^44^ Shanghai Treatgut Biotechnology Co., Ltd Shanghai China; ^45^ Senior Department of Tuberculosis the Eighth Medical Center of PLA General Hospital Beijing China; ^46^ School of Basic Medical Sciences Henan University Kaifeng China; ^47^ Beijing Bio Huaxing Gene Technology Co., LTD Beijing China; ^48^ School of Life Science, Beijing Institute of Technology Beijing China; ^49^ Wuhan University Wuhan China; ^50^ School of Pharmacy Gannan Medical University Ganzhou China; ^51^ School of Laboratory Medicine Hubei University of Chinese Medicine Wuhan China; ^52^ Jianghan university Wuhan China; ^53^ College of Animal Sciences Zhejiang University Hangzhou China; ^54^ College of Pharmacy Hubei University of Chinese Medicine Wuhan China; ^55^ Department of Gastroenterology Second Affiliated Hospital of Zhejiang University School of Medicine Hangzhou Zhejiang China; ^56^ Department of Urology, Changhai Hospital Naval Medical University, Second Military Medical University Shanghai China; ^57^ College of Food Science and Engineering Henan University of Technology Zhengzhou China; ^58^ State Key Laboratory of Microbial Technology Shandong University Qingdao China; ^59^ Kunming Institute of Zoology, Chinese Academy of Sciences Kunming China; ^60^ College of Advanced Agriculture Sciences Zhejiang Agricultural and Forestry University Hangzhou China; ^61^ State Key Laboratory of Synthetic Biology, Frontiers Science Center for Synthetic Biology (Ministry of Education), and School of Synthetic Biology and Biomanufacturing Tianjin University Tianjin China; ^62^ Department of Animal Science and Technology, College of Animal Sciences Zhejiang University Hangzhou China; ^63^ Zhejiang Agriculture and Forestry University Hangzhou China; ^64^ Hubei University of Chinese Medicine Wuhan China; ^65^ Chinese Evidence‐based Medicine Center, West China Hospital Sichuan University Chengdu China; ^66^ Guizhou Provincial Institute of Biology, Guizhou Academy of Sciences Guiyang China; ^67^ National key laboratory of agricultural microbiology, Key Laboratory of Environment Correlative Dietology, College of Biomedicine and Health Huazhong Agricultural University, Shenzhen Institute of Nutrition and Health, Huazhong Agricultural University Wuhan China; ^68^ College of Agriculture and Biotechnology Zhejiang University Hangzhou China; ^69^ Henan Agricultural University Zhengzhou China; ^70^ College of Biological Engineering Qingdao University of Science and Technology Qingdao China; ^71^ Tianjin Institute of Industrial Biotechnology, Chinese Academy of Sciences Tianjin China; ^72^ Department of Neurosurgery, Department of Neuro‐oncological Surgery, The National Key Clinical Specialty, The Engineering Technology Research Center of Education Ministry of China on Diagnosis and Treatment of Cerebrovascular Disease, Guangdong Provincial Key Laboratory on Brain Function Repair and Regeneration, The Neurosurgery Institute of Guangdong Province, Zhujiang Hospital Southern Medical University Guangzhou China; ^73^ Department of Cardiology, Center for Translational Medicine Research, Zhujiang Hospital Southern Medical University Guangzhou China; ^74^ Institute of Microbial Technology Shandong University Qingdao China; ^75^ Department of Clinical Laboratory Shandong Provincial Hospital Affiliated to Shandong First Medical University Jinan China; ^76^ Hubei Key Laboratory of Hepato‐Pancreato‐Biliary Diseases Wuhan China; ^77^ Clinical Laboratory of Integrative Medicine The First Affiliated Hospital of Dalian Medical University Dalian China; ^78^ School of Medicine Shanghai Jiao Tong University Shanghai China; ^79^ Department of Gastroenterology Institute of Digestive Disease, Guangxi Academy of Medical Sciences, the People's Hospital of Guangxi Zhuang Autonomous Region Nanning China; ^80^ College of Life Sciences Shandong Agricultural University Tai'an China; ^81^ XiangHu Laboratory Hangzhou China; ^82^ Medical Research Center Southern University of Science and Technology Hospital Shenzhen China; ^83^ Collaborative Innovation Center for Zhejiang Marine High‐Efficiency and Healthy Aquaculture Ningbo University, Ningbo China; ^84^ Institute of Farmland Irrigation, Chinese Academy of Agricultural Sciences Xinxiang China; ^85^ College of Veterinary Medicine Nanjing Agricultural University Nanjing China; ^86^ Biotree Metabolomics Technology Research Center Shanghai China; ^87^ State Key Laboratory of Seed Innovation, Institute of Genetics and Developmental Biology, Chinese Academy of Sciences Beijing China; ^88^ National Key Laboratory of Intelligent Tracking and Forecasting for Infectious Diseases, National Institute for Communicable Disease Control and Prevention, Chinese Center for Disease Control and Prevention Beijing China; ^89^ College of Biology Hunan University Changsha China; ^90^ Institute of Grassland Research, Chinese Academy of Agricultural Sciences Hohhot Inner Mongolia Autonomous Region China; ^91^ Oncology Department of Integrative Medicine China‐Japan Friendship Hospital Beijing China; ^92^ Chengdu University of Traditional Chinese Medicine Chengdu China; ^93^ Chinese Academy of Medical Sciences and Peking Union Medical College Beijing China; ^94^ National‐Local Joint Engineering Research Center for Biomass Refining and High‐Quality Utilization, Institute of Urban and Rural Mining Changzhou University Changzhou China; ^95^ Department of Toxicology and Health Inspection and Quarantine, School of Public Health Tianjin Medical University Tianjin China; ^96^ Zhejiang Cancer Hospital, Hangzhou Institute of Medicine (HIM) Chinese Academy of Sciences Hangzhou China; ^97^ Center for RNA Medicine, the Fourth Affiliated Hospital of School of Medicine, International School of Medicine, International Institutes of Medicine Zhejiang University Yiwu China; ^98^ College of Environmental Science and Engineering Ocean University of China Qingdao China; ^99^ State Key Laboratory of Crop Stress Resistance and High‐Efficiency Production, College of Life Sciences Northwest A&F University Yangling China; ^100^ Research Centre of Basic Medical Sciences, Medical College Qinghai University Xining China; ^101^ College of Computer Science and Technology National University of Defense Technology Changsha China; ^102^ State Key Laboratory of Maize Bio‐breeding and Key Laboratory of Maize Biology and Genetic Breeding, Ministry of Agriculture and Rural Affairs China Agricultural University Beijing China; ^103^ Department of Medicine and Therapeutics The Chinese University of Hong Kong Hong Kong SAR China; ^104^ College of Animal Science and Technology Northwest A&F University Yangling China; ^105^ Microbiology Department of Basic Medical College of Xinjiang Medical University Xinjiang Medical University Urumqi China; ^106^ Department of Interventional Radiology, Zhongshan Hospital Fudan University Shanghai China; ^107^ State Key Laboratory of Tropical Crop Breeding, Shenzhen Branch, Guangdong Laboratory of Lingnan Modern Agriculture, Key Laboratory of Synthetic Biology, Ministry of Agriculture and Rural Affairs, Agricultural Genomics Institute at Shenzhen, Chinese Academy of Agricultural Sciences Shenzhen Guangdong China; ^108^ Department of Psychiatry the First Affiliated Hospital of Chongqing Medical University, key Laboratory of Major Brain Disease and Aging Research (Ministry of Education), Psychiatric Center of Chongqing Medical University the First Affiliated Hospital Chongqing China; ^109^ Department of Clinical Laboratory The Affiliated Li Huili Hospital of Ningbo University Ningbo China; ^110^ College of Preventive Medicine Army Medical University Chongqing China; ^111^ Department of Pharmacology, School of Basic Medical Sciences Wuhan University Wuhan China; ^112^ Key Laboratory of Pesticide & Chemical Biology of Ministry of Education, School of Life Sciences Central China Normal University Wuhan China; ^113^ Institute for Math & AI Wuhan University Wuhan China; ^114^ China Agricultural Univsersity Beijing China; ^115^ School of Public Health Southeast University Nanjing China; ^116^ School of Information Science and Technology Beijing Forestry University Beijing China; ^117^ Department of Neonatology Affiliated Shenzhen Women and Children's Hospital (Longgang) of Shantou University Medical College (Longgang District Maternity & Child Healthcare Hospital of Shenzhen City) Shenzhen China; ^118^ National Center for Integrative Medicine, China Japan Friendship Hospital Beijing China; ^119^ Department of Biomedical Informatics, School of Life Sciences Central South University Changsha China; ^120^ State Key Laboratory of Crop Stress Resistance and High‐Efficiency Production, College of Plant Protection Northwest A&F University Yangling China; ^121^ College of Agriculture Guizhou University Guiyang China; ^122^ MOE Key Laboratory of Metabolism and Molecular Medicine, Department of Biochemistry and Molecular Biology, School of Basic Medical Sciences Fudan University Shanghai China; ^123^ Key Laboratory of Virology and Biosafety and National Virus Resource Center, Wuhan Institute of Virology, Chinese Academy of Sciences Wuhan China; ^124^ University of Chinese Academy of Sciences Beijing China; ^125^ JinFeng Laboratory Chongqing China; ^126^ State Key Laboratory of Trauma and Chemical Poisoning; Chongqing Key Laboratory of Hematology and Microenvironment Chongqing China; ^127^ The Second Affiliated Hospital of Zhejiang Chinese Medical University Hangzhou China; ^128^ School of Pharmaceutical Sciences, State Key Laboratory of Antiviral Drugs, Pingyuan Laboratory Zhengzhou University Zhengzhou China; ^129^ School of Chinese Medicine, Medical Department Jianghan University Wuhan China; ^130^ Department of pathology Beilun People's Hospital Ningbo China; ^131^ Guangxi Key Laboratory of Animal Breeding, Disease Control and Prevention, College of Animal Science and Technology Guangxi University Nanning China; ^132^ College of medical information and artificial intelligence Shandong First Medical University Jinan China; ^133^ State Key Laboratory of Drug Research, Shanghai Institute of Materia Medica, Chinese Academy of Sciences Shanghai China; ^134^ Institute of Plant Protection Research Henan Academy of Agricultural Sciences Zhengzhou China; ^135^ School of Chinese Medicine Hong Kong Baptist University Hong Kong China; ^136^ Laboratory of Integrative Medicine First Affiliated Hospital of Dalian Medical University Dalian China; ^137^ Department of Orthopedic Surgery the First Hospital of China Medical University Shenyang China; ^138^ Shandong university of traditional Chinese medicine Jinan China; ^139^ College of Resources and Environment University of Chinese Academy of Sciences Beijing China; ^140^ Precision Oncology and Intelligent Theranostics Laboratory, Department of Pediatric Hematology and Oncology Children's Hospital of Chongqing Medical University Chongqing China; ^141^ Research Center for Mathematics and Interdisciplinary Sciences, Frontiers Science Center for Nonlinear Expectations (Ministry of Education) Shandong University Qingdao China; ^142^ College of fisheris Huazhong Agricultural University Wuhan China; ^143^ Affiliated Women's Hospital of Jiangnan University Wuxi China; ^144^ Department of Microbiology, LKS Faculty of Medicine The University of Hong Kong Hong Kong SAR China; ^145^ School of Public Health (Shenzhen) Sun Yat‐sen University Shenzhen China; ^146^ Xiangya School of Basic Medical Sciences Central South University Changsha China; ^147^ The First Department of Gastroenterology The First Affiliated Hospital of Xinjiang Medical University Urumqi China; ^148^ Department of Orthopaedics The Second Hospital of Shandong University, Cheeloo College of Medicine, Shandong University Jinan China; ^149^ State Key Laboratory of Herbage Improvement and Grassland Agro‐ecosystems, and College of Ecology Lanzhou University Lanzhou China; ^150^ College & Hospital of Stomatology Anhui Medical University, Key Lab. of Oral Diseases Research of Anhui Province Hefei China; ^151^ First Clinical Medical College, Shandong University of Traditional Chinese Medicine, The Department of Pediatrics Affiliated Hospital of Shandong University of Traditional Chinese Medicine Jinan China; ^152^ College of Veterinary Medicine South China Agriculture University Guangzhou China; ^153^ Interdisciplinary Science Center, State Key Laboratory of Animal Biodiversity Conservation and Integrated Pest Management, Institute of Zoology, Chinese Academy of Sciences Beijing China; ^154^ Institute of Digestive Disease, Department of Medicine and Therapeutics, State Key Laboratory of Digestive Disease The Chinese University of Hong Kong Hong Kong SAR China; ^155^ College of Biological Engineering Henan University of Technology Zhengzhou China; ^156^ Shenzhen Branch, Guangdong Laboratory of Lingnan Modern Agriculture, Genome Analysis Laboratory of the Ministry of Agriculture and Rural Affairs, Agricultural Genomics Institute at Shenzhen, Chinese Academy of Agricultural Sciences Shenzhen China; ^157^ Affiliated Hospital of Jiangnan University Nantong China; ^158^ Institute of Reproductive Health, Tongji Medical College Huazhong University of Science and Technology Wuhan China; ^159^ Zhongnan hospital of Wuhan university Wuhan China; ^160^ College of Veterinary Medicine Northeast Agricultural University Harbin China; ^161^ Beijing Institute of Biotechnology Beijing China; ^162^ Department of Rheumatology Fujian Medical University Union Hospital Fuzhou China; ^163^ Cancer Institute, Suzhou Medical College Soochow University Suzhou China; ^164^ Division of Gastroenterology, Union Hospital, Tongji Medical College Huazhong University of Science and Technology Wuhan China; ^165^ The Children's Hospital Zhejiang University School of Medicine, National Clinical Research Center for Child Health Hangzhou China; ^166^ State Key Laboratory of Green Pesticide Central China Normal University Wuhan China; ^167^ The Second Affiliated Hospital of Soochow University, Cancer Institute, Suzhou Medical College, Soochow University Suzhou China; ^168^ Donghai County People's Hospital (Affiliated Kangda College of Nanjing Medical University) Lianyungang China; ^169^ MOE Frontier Science Centre for Precision Oncology University of Macau Macau SAR China; ^170^ Department of Oncology Zhujiang Hospital of Southern Medical University Guangzhou China; ^171^ Department of Geriatrics Affiliated Hospital of Hubei University of Chinese Medicine, Hubei University of Chinese Medicine Wuhan China

## Abstract

The *iMeta* Conference 2025, part of the *iMeta* Conference series, themed “Creating High‐Impact International Journals,” held at the Huangjiahu Campus of Hubei University of Chinese Medicine from August 23rd to 25th, 2025, and focused on frontier topics such as microbiology, medicine, traditional Chinese medicine, botany, and research career development. The event aimed to support the development of researchers and strengthen the impact of academic journals. Through invited reports, thematic seminars, and poster presentations, the conference highlighted hot topics including multi‐omics technologies, microbe‐host interactions, AI‐assisted research, live biotherapeutic products, and the modernization of traditional Chinese medicine. The event demonstrated the innovative momentum of interdisciplinary integration and technological convergence, providing an international platform for academic exchange and laying a foundation for building an innovative scientific research ecosystem and enhancing the global influence of Chinese academic journals.

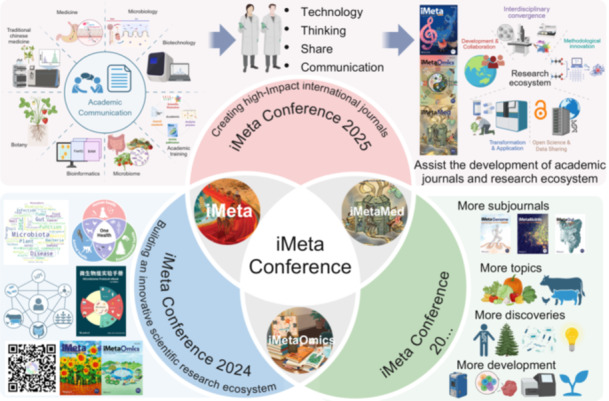

From August 23 to 25, 2025, the *iMeta* Conference 2025 was successfully held at the Huangjiahu Campus of Hubei University of Chinese Medicine. Hosted by the Editorial Office of *iMeta*, Hubei University of Chinese Medicine, Hubei Shizhen Laboratory, and others, the event invited more than 70 academicians, distinguished/excellent young scholars, and attracted over 400 international participants all over the world. In the past 5 years, China has developed 24 highly impactful journals (impact factor/IF > 20), reaching half the number of such journals in the UK and the US, thus establishing the “third pole” of scholarly publishing. The conference emphasized the importance of uniting domestic disciplinary strengths to establish a globally influential editorial and journal system (Figure 1), aiming to provide alternatives to top international journals such as *Cell*, *Nature*, and *Science*. Its sub‐journals, *iMetaOmics* (expected IF > 10) [1] and *iMetaMed* (expected IF > 15), are also dedicated to building international influence. The long‐term goal is to establish 10 sub‐journals with IF > 10 within 8 years, forming a “Chinese version” to supplementary/option with the *Cell* series of biomedical journals.

**Figure 1:**
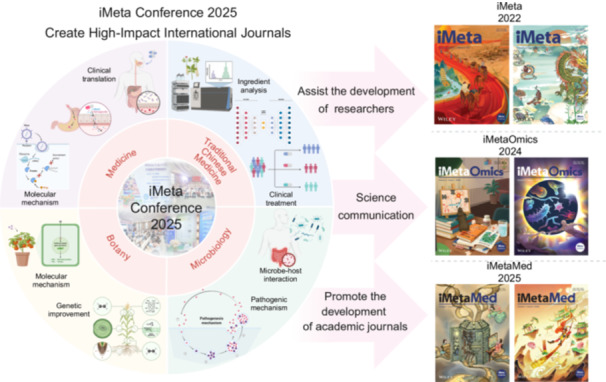
Overview of the themes and meaning of *iMeta* Conference 2025.

Since its inception in 2022, the *iMeta* Conference has been held in Qingdao, Beijing, and Shenzhen [2]. The 2025 session continued its mission of tracking disciplinary frontiers, promoting scientific development, and advancing international collaboration.

## The opening ceremony

The opening ceremony, chaired by Yongxin Liu (Executive Editor‐in‐Chief, *iMeta*), featured welcome addresses by President Gang Chen (Hubei University of Chinese Medicine) and Professor Shuangjiang Liu (Editor‐in‐Chief, *iMeta*). During the ceremony, a strategic cooperation agreement was signed between the *iMeta* Editorial Office and the university, marking a milestone in jointly enhancing the international influence of Chinese academic journals.

At the plenary invited report session, academician Zixin Deng from Shanghai Jiao Tong University delivered a talk entitled “Big Health Industry and Synthetic Biology” He systematically elaborated on the core role of synthetic biology as a disruptive technological engine in reshaping the biotechnology landscape and empowering the upgrading of the big health industry, vividly demonstrating the strong application and translational potential of synthetic biology [3]. Academician Peiwu Li from the Oil Crops Research Institute of the Chinese Academy of Agricultural Sciences gave a report entitled “ARC Biological Coupling Technology for Quality Improvement and Nitrogen Fixation in Soybean and Oil Crops” He introduced the experience in inventing ARC microbial agents and applying them on a large scale [4]. Academician Mingyong Xie from Nanchang University presented a report entitled “Health Benefits of Plant‐Based Lactic Acid Bacteria Fermented Foods” He briefly reviewed the current research status of plant‐based lactic acid bacteria fermented foods in China and abroad, and discussed future development trends in this field [5].

In addition, Professor Yizhun Zhu from Macau University of Science and Technology delivered a report entitled “Innovative Drug Development and the Empowerment of Transcriptomics” showcasing the latest breakthroughs in metabolic disease research using transcriptomics technology [6]. Professor Shuangjiang Liu from the Chinese Academy of Sciences gave a report entitled “From Intestinal Bacteria to Functional Probiotics/Live Biotherapeutic Products‐A Case Study of Christensenellads” He highlighted the species diversity, multiple interactions between host and gut Christensenella species, and their implications for the development of probiotics/LBPs [7]. Director Wei Xu from Treatgut Biotechnology Co., Ltd introduced the company's focus on tackling bottleneck issues in the human microecological medical industry from both technological and product perspectives. Finally, Xiaodong Tao, President of Xunfei Healthcare Co., Ltd., pointed out that artificial intelligence technology, by mining potential disease patterns through deep learning algorithms and assisting diagnostic decision‐making, will significantly promote the construction of a precision diagnosis and treatment system.

## Conference reports

The conference featured four plenary sessions on Basic Medical Sciences, Microbiome Research, Bioinformatics, and Biomedicine, collectively showcasing the latest advances in life science innovation driven by interdisciplinary integration and cutting‐edge technologies.

The Basic Medical Sciences session focused on multi‐omics, nanotechnology, and microbial regulation, covering key areas such as tumor microenvironment analysis, applications of spatial transcriptomics, and epigenetic regulation, emphasizing the potential of technological integration and clinical translation [8]. The Microbiome Research session systematically explored the interaction mechanisms of multiple systems, including the gut–brain axis and gut–joint axis, and demonstrated innovative applications of microbiome interventions in disease treatment and health management [9]. The Bioinformatics session highlighted methodological innovations in multi‐omics and artificial intelligence for microbiomes, viromes, and host–pathogen interactions, presenting a new data‐driven research paradigm [10]. The Biomedicine session delved into disease mechanisms from the perspectives of microbial metabolites, epigenetic regulation, and multi‐omics integration, while also envisioning future prospects for precision medicine and nutritional interventions. Together, the four sessions reflected full‐chain innovation from fundamental discoveries to translational research, capturing the prevailing trend in life sciences of interdisciplinary integration, technological convergence, and clinical application.

## Researchers development forum

The Researchers Development Forum focused on the academic development and career advancement of young researchers, addressing three themes: project applications, interdisciplinary innovation, and research experience sharing.

In the project application section, Chang Liu (Shandong University) and Dong Zhang (Lanzhou University) shared experiences and strategies regarding applications for the National Natural Science Foundation of China, respectively, providing practical advice for young scholars. Several talks highlighted interdisciplinary integration and applications of cutting‐edge technologies: Jinyang Zhang (Institute of Zoology, Chinese Academy of Sciences) presented precise analysis and functional exploration of circular RNA [11]. Feng Xu (Shandong First Medical University) discussed the latest applications of artificial intelligence in biomedicine, and Motong Chen (Institute of Microbiology, Guangdong Academy of Sciences) showcased research on risk identification of foodborne Listeria monocytogenes.

The Traditional Chinese Medicine (TCM) section demonstrated the integration of tradition with modern science [12]. Ling Li (West China Hospital, Sichuan University) introduced systematic evidence‐based approaches for complex TCM interventions; Chun Li (Beijing University of Chinese Medicine) shared her research journey on TCM syndromes; Xin Luan (Shanghai University of Chinese Medicine) and Honglei Jin (Guangzhou University of Chinese Medicine) presented innovative paths for TCM modernization, focusing on active compound mechanisms and integration of life sciences with TCM resource science. In addition, Wenting Li (Henan Agricultural University) shared her research trajectory from basic studies to molecular breeding under the theme “Accumulation, Inheritance, and Growth,” highlighting continuity and breakthroughs in agricultural biotechnology.

The forum addressed both practical needs for project applications and talent development, while emphasizing the importance of interdisciplinary collaboration and technology convergence in advancing biomedicine and TCM, providing a valuable platform for young researchers to exchange ideas and gain inspiration.

## Researchers advancement forum

The Researchers Advancement Forum invited academician Meilin Jin and other renowned experts for on‐site commentary. Centered on “Interdisciplinary Innovation in Precision Medicine, Intelligent Computing, and Microbiomics” the forum covered several frontier research directions. Hua You (Chongqing Medical University) introduced the construction of a precision treatment system for childhood leukemia; Zhemin Zhou (Soochow University) discussed computational pathogen genomics in infectious disease prevention and control; Shaoxing Dai (Kunming University of Science and Technology) presented how omics and AI can drive drug discovery; Qi Su (The Chinese University of Hong Kong) reported on probiotic regulation of insomnia; and Zhuang Li (Southern Medical University) proposed microbiome‐derived biomarkers for inflammatory diseases.

After the coffee break, Kun Jiang (Shandong University) analyzed bacterial toxin‐mediated mechanisms in microbiota–environment interactions; Feng Li (Tianjin University) discussed the design and biomanufacturing of electroactive microbial cell factories; Tong Chen (China Academy of Chinese Medical Sciences) shared strategies for integrating TCM big data resources; and Tao Wen (Nanjing Agricultural University) explored new approaches for regulating microbial populations in crop soil‐borne diseases via rhizosphere chemical signals. The forum underscored interdisciplinary integration and original innovation, focusing on scientific problem refinement, research design, and innovation in grant proposals, providing an in‐depth exchange platform for advancing highly impact research careers.

## The traditional chinese medicine summit forum

The TCM Summit Forum brought together leading experts to discuss TCM modernization and interdisciplinary innovation. Professor Hongcai Shang (Beijing University of Chinese Medicine) proposed the concept of “digital and intelligent empowerment” highlighting AI and big data applications in evidence‐based TCM research. Professor Peng Cao (Nanjing University of Chinese Medicine), in his talk “Retracing the Path of ‘Shennong Tastes a Hundred Herbs’”, introduced the discovery of active substances in fresh TCM and their value in modern drug development.

Subsequently, Professor Yongjun Wang (Shanghai University of Chinese Medicine) discussed the systems biology of the “Kidney Essence” theory in preventing and treating chronic bone‐marrow‐brain diseases, while Professor Jiaxu Chen (Beijing University of Chinese Medicine) used the classic prescription Xiaoyao Powder to analyze the TCM research paradigm combining “disease‐syndrome‐prescription” advancing modern interpretations of TCM formula theory. This forum highlighted the integration of TCM theory with modern science, showing significant progress in chronic disease prevention, active substance discovery, and research paradigm transformation, while providing a high‐level dialogue platform for building an independent TCM knowledge system.

## Frontier forum on medicine

The Frontier Forum on Medicine focused on disease prevention, microbe‐host interactions, and interdisciplinary innovation, presenting progress from basic research to clinical translation.

Key topics included: Professor Jingyuan Fu (University of Groningen, Netherlands) reported on microbial and metabolic changes in acute coronary syndrome and its recovery phase; Wenhua Liang (Guangzhou Medical University) proposed new strategies for early lung cancer prevention; Canzhao Liu (Southern Medical University) revealed the role of PDZ proteins in vascular remodeling; Shuofeng Yuan (University of Hong Kong) explored broad‐spectrum antiviral targets; and Kaibin Shi (Capital Medical University) and Shanshan Liu (Central South University) discussed neurodegeneration in multiple sclerosis/stroke and therapeutic targets for pulmonary fibrosis.

Another focus was the microbiome and precision therapies. Jinyao Liu (Shanghai Jiao Tong University) presented precision delivery technologies for LBPs; Xiaolong He (Southern Medical University) focused on mining functional enzymes in the gut microbiome; Hui Wang (Wuhan University) revealed the role of maternal microbiota metabolite daidzein in fetal‐originated osteoporosis prevention; and Xiang Zhang (The Chinese University of Hong Kong) presented the latest findings on metabolic‐associated fatty liver disease. Ming Xu (Peking University) emphasized the importance of interdisciplinary integration in advancing medical innovation. The forum demonstrated the synergy between molecular research and clinical strategies, underscoring multidisciplinary collaboration in tackling major diseases [13].

## Frontier forum on botany

The Botany Frontier Forum focused on crop stress resistance, signal transduction, and genetic improvement [14]. Scholars shared insights from molecular mechanisms to breeding applications: Hongtao Liu (Shenzhen University) revealed the functional differentiation of blue light receptors; Xiaojie Wang and Qingmei Guan (Northwest A&F University) presented new disease‐resistance breeding strategies; Fei Zhang (Huazhong Agricultural University) established a model system for citrus thorn development [15]; and Jun Wu (Nanjing Agricultural University) and Zhiyong Liu (Chinese Academy of Sciences) discussed pear genome achievements and precise wheat resistance gene design.

Other highlights included Hongning Tong (Chinese Academy of Agricultural Sciences) on tubulin phase transitions in rice BR signaling [16]; Hao Lin (Chinese Academy of Agricultural Sciences) on alfalfa genetic resource mining; Weibin Song (China Agricultural University) on the genetic basis of maize yield traits; and Shi Xiao (Sun Yat‐sen University) on hypoxia sensing and plant stress adaptation. The forum highlighted full‐chain innovation from gene discovery to breeding applications, underscoring plant science's role in food security and sustainable agriculture.

## Frontier forum on microbiology

The Microbiology Frontier Forum addressed pathogenic and environmental microorganisms as well as host–microbe interactions [17]. Linqi Wang (Institute of Microbiology, Chinese Academy of Sciences) revealed a new mechanism of host‐driven fungal resistance [18]; Cuihua Liu discussed host–pathogen strategies in tuberculosis; Zhengguo He (Wuhan University) analyzed the potential of mycobacteriophages in research and clinical translation; and Xihui Shen (Northwest A&F University) presented bacterial cross‐kingdom recognition of fungi [19].

In environmental microbiology and synthetic biology, Hongzhi Tang (Shanghai Jiao Tong University) highlighted applications of synthetic biology in environmental remediation [20]; Dawei Zhang (Tianjin Institute of Industrial Biotechnology, CAS) proposed vitamin biosynthesis strategies based on DBTL (Design‐Build‐Test‐Learn) and LDBA (Laboratory Automation) cycles; Fuhe Chen (University of Hong Kong) explored “long COVID” mechanisms; Yancong Zhang (Chinese Academy of Agricultural Sciences) introduced functional predictions of microbial “dark matter”; Weihua Chen (Huazhong University of Science and Technology) applied coculture techniques to gut microbiota; and Dingjie Wang (Wuhan University) developed third‐generation sequencing for accurate isoform quantification. The forum reflected the expansion of microbiology towards systematization, intelligentization, and applied sciences.

## Biotechnology session

The Biotechnology Session showcased how innovations in sequencing, AI, and microbiome research are reshaping life sciences. Juan Shen (BGI‐Research) used single‐cell spatiotemporal omics to study host–microbe interactions in germ‐free mice; Hu Chen (Wuhan Benagen Technology) reviewed nanopore sequencing progress and challenges [21]; Qiyi Liang (Beijing Bio Huaxing) demonstrated AI‐driven genomic breeding; Lishan Zhang (Xiamen Treatgut Biotechnology) presented live biotherapeutic development strategies for ulcerative colitis; and Defeng Bai (Agricultural Genomics Institute at Shenzhen) launched EasyMetagenome, a standardized metagenomic pipeline. The session highlighted the complete chain of biotech innovation from tools to clinical translation [22].

## Scientific research training session

The Scientific Research Training Session focused on scientific image production, data visualization, and international journal development. Yuanyuan Bei (PaperArtist) outlined standards for SCI illustrations; Tong Chen (China Academy of Chinese Medical Sciences) demonstrated efficient charting with ImageGP; and Dan Song (University of Chinese Academy of Sciences) discussed journal‐building strategies using “Cell Proliferation” as an example. This session equipped researchers with tools and strategies to enhance academic communication and international competitiveness [23, 24].

## Postdoctor and graduate forum

The Postdoctoral and Graduate Forum showcased young scholars' research on microbiomes, multi‐omics, and host‐disease interactions. Topics included microbial metabolism regulation, STAT1 lactylation in gastric cancer, engineered bacteria antitumor strategies, and the impact of bile acids/butyrate on metabolic diseases. New tools and resources, such as long‐read metagenomics, global wildlife microbiome databases, MicrobiomeStatPlots, and digital twin models, were introduced. The forum highlighted the creativity and technical diversity of early‐career researchers.

## Abstracts and poster exhibition

The conference featured an abstract collection and poster exhibition, with over 40 cutting‐edge studies from domestic and international institutions. Topics spanned microbiomics, precision medicine, TCM modernization, and biotechnology. The poster session provided an interactive platform for young researchers, fostering collaboration and innovation.

## Summary

The *iMeta* Conference 2025 was successfully held at Hubei University of Chinese Medicine from August 23–25, bringing together more than 400 scholars worldwide and over 70 academicians, distinguished/excellent young scholars. Discussions centered on advancing highly impact international journals within China's independent knowledge system. The event systematically explored the path of Chinese journals from “following” to “leading” emphasizing global impact through interdisciplinary integration, industry‐academia collaboration, and international cooperation. With multiple forums covering microbiomes, medicine, bioinformatics, TCM, botany, and microbiology, the conference highlighted frontier research such as microbe–host interactions, multi‐omics, live biotherapeutics, and AI‐assisted studies. Distinguished academicians delivered invited talks on synthetic biology, agricultural microbiology, lactic acid fermentation, and the health industry, showcasing scientific empowerment from basic research to applications. The conference not only provided a highly impact exchange platform but also charted a path for Chinese journals to achieve global leadership.

## AUTHOR CONTRIBUTIONS

**Zhihao Zhu, Lin Zhang, Xiaofang Yao, Meiyin Zeng, Yao Wang, Hao Luo, Yuanping Zhou, Tianyuan Zhang, Jiani Xun, Defeng Bai, Haifei Yang, Shanshan Xu, Yang Zhou, Yunyun Gao, Jinbo Xu, Wei Han, ZiAng Shen and Bangzhou Zhang**: Writing—original draft; visualization; data curation; investigation, project administration. **Suyin Feng, Shuangxia Jin, Chuanxing Xiao, Ziheng Wang, Peng Luo, Tong Chen, Gang Chen, Yong‐Xin Liu**: Conceptualization, supervision, funding acquisition, project administration. All authors: Writing—review and editing. All authors have read the final manuscript and approved it for publication.

## CONFLICT OF INTEREST STATEMENT

The authors declare no conflicts of interest. Shuang‐Jiang Liu is the Editor‐in‐Chief of *iMeta*; Yong‐Xin Liu and Tong Chen are Executive Editors of iMeta.

## ETHICS STATEMENT

No animals or humans were involved in this study.

## Supporting information


**Table S1:** *iMeta* conference 2025 meeting schedule in Chinese and English.

## Data Availability

The data that support the findings of this study are available in meeting website: http://www.imeta.science/meeting/2025. Supplementary materials (table, graphical abstract, slides, videos, Chinese translated version, and update materials) may be found in the online DOI or *iMeta* Science http://www.imeta.science/.
